# Developing a cooperative multicenter study in Latin America: Lessons learned from the Latin American Study of Nutrition and Health Project

**DOI:** 10.26633/RPSP.2017.111

**Published:** 2017-12-19

**Authors:** Mauro Fisberg, Irina Kovalskys, Georgina Gómez Salas, Rossina Gabriella Pareja Torres, Martha Cecilia Yépez García, Lilia Yadira Cortés Sanabria, Marianella Herrera-Cuenca, Attilio Rigotti, Viviana Guajardo, Ioná Zalcman Zimberg, Agatha Nogueira Previdelli, Luis A. Moreno, Michael Pratt, Berthold Koletzko, Katherine L. Tucker, ELANS Study Group

**Affiliations:** 1 Instituto Pensi, Fundação Jose Luiz Egydio Setubal Hospital Infantil Sabara São Paulo Brazil Instituto Pensi, Fundação Jose Luiz Egydio Setubal, Hospital Infantil Sabara, São Paulo, Brazil.; 2 Committee on Nutrition and Wellbeing International Life Science Institute Buenos Aires Argentina Committee on Nutrition and Wellbeing, International Life Science Institute, Buenos Aires, Argentina.; 3 Escuela de Medicina, Universidad de Costa Rica Departamento de Bioquímica San José Costa Rica Departamento de Bioquímica, Escuela de Medicina, Universidad de Costa Rica, San José, Costa Rica.; 4 Instituto de Investigación Nutricional Instituto de Investigación Nutricional Lima Peru Instituto de Investigación Nutricional, Lima, Peru.; 5 Colegio de Ciencias de la Salud Universidad San Francisco de Quito Quito Ecuador Colegio de Ciencias de la Salud, Universidad San Francisco de Quito, Quito, Ecuador.; 6 Departamento de Nutrición y Bioquímica Pontificia Universidad Javeriana Bogotá Colombia Departamento de Nutrición y Bioquímica, Pontificia Universidad Javeriana, Bogotá, Colombia.; 7 Centro de Estudios del Desarrollo Universidad Central de Venezuela/Fundación Bengoa Caracas Venezuela Centro de Estudios del Desarrollo, Universidad Central de Venezuela/Fundación Bengoa, Caracas, Venezuela.; 8 Departamento de Nutrición Diabetes y Metabolismo, Centro de Nutrición Molecular y Enfermedades Crónicas, Escuela de Medicina, Pontificia Universidad Católica Santiago Chile Departamento de Nutrición, Diabetes y Metabolismo, Centro de Nutrición Molecular y Enfermedades Crónicas, Escuela de Medicina, Pontificia Universidad Católica, Santiago, Chile.; 9 Departamento de Psicobiologia Universidade Federal de São Paulo São Paulo Brazil Departamento de Psicobiologia, Universidade Federal de São Paulo, São Paulo, Brazil.; 10 Faculdade de Ciências Biológicas e da Saúde Universidade São Judas Tadeu São Paulo Brazil Faculdade de Ciências Biológicas e da Saúde, Universidade São Judas Tadeu, São Paulo, Brazil.; 11 Growth, Exercise, Nutrition and Development Research Group Instituto Agroalimentario de Aragón, Instituto de Investigación Sanitaria Aragón, University of Zaragoza Zaragoza Growth, Exercise, Nutrition and Development Research Group, Instituto Agroalimentario de Aragón, Instituto de Investigación Sanitaria Aragón, Centro de Investigación Biomédica en Red Fisiopatología de la Obesidad y Nutrición, University of Zaragoza, Zaragoza.; 12 Nutrition and Health Sciences Program, Hubert Department of Global Health Rollins School of Public Health, Emory University United States of America Nutrition and Health Sciences Program, Hubert Department of Global Health, Rollins School of Public Health, Emory University, United States of America.; 13 Division of Metabolic and Nutritional Medicine Dr. Von Hauner Children’s Hospital, University of Munich Medical Center Munich Germany Division of Metabolic and Nutritional Medicine, Dr. Von Hauner Children’s Hospital, University of Munich Medical Center, Munich, Germany.; 14 Department of Clinical Laboratory and Nutritional Sciences University of Massachusetts, Lowell, Massachusetts United States Department of Clinical Laboratory and Nutritional Sciences, University of Massachusetts, Lowell, Massachusetts, United States.; 15 The ELANS Study Group The ELANS Study Group. For a complete list of members, see the Acknowledgements.

**Keywords:** Multicenter study, nutritional surveillance, nutrition surveys, Latin America, Estudio multicéntrico, vigilancia nutricional, encuestas nutricionales, América Latina

## Abstract

This report examines the challenges of conducting a multicenter, cross-sectional study of countries with diverse cultures, and shares the lessons learned. The Latin American Study of Nutrition and Health (ELANS) was used as a feasibility study involving the most populous cities of eight countries in Latin America (Argentina, Brazil, Chile, Colombia, Costa Rica, Ecuador, Peru, and Venezuela) in 2014–2015, about 40% of the population of the Americas. The target sample included 9 000 individuals, 15–65 years of age, and was stratified by geographic location (only urban areas), gender, age, and socioeconomic status.

Six principal challenges were identified: team structuring and site selections; developing a single protocol; obtaining ethic approvals; completing simultaneous fieldwork; ensuring data quality; and extracting data and maintaining consistency across databases. Lessons learned show that harmonization, pilot study, uniformity of procedures, high data quality control, and communication and collaboration across sites are imperative. Barriers included organizational complexity, recruitment of collaborators and research staff, institutional cooperation, development of infrastructure, and identification of resources. Consensus on uniform measures and outcomes and data collection methodology, as well as a plan for data management and analysis, communication, publication, and dissemination of study results should be in place prior to beginning fieldwork. While challenging, such studies offer great potential for building a scientific base for studies on nutrition, physical activity, and other health topics, while facilitating comparisons among countries.

## RESUMEN

En este informe se examinan los retos de llevar a cabo un estudio transversal multicéntrico en países con culturas diversas y se transmiten las enseñanzas extraídas. Se usó el Estudio Latinoamericano de Nutrición y Salud (ELANS) como un estudio de factibilidad realizado en el período 2014–2015, que incluyó las ciudades más populosas de ocho países de América Latina (Argentina, Brasil, Chile, Colombia, Costa Rica, Ecuador, Perú y Venezuela) y abarcó a cerca de 40% de la población de la Región de las Américas. La muestra establecida incluyó a 9 000 personas de 15 a 65 años de edad, y fue estratificada según la ubicación geográfica (solo zonas urbanas) y por sexo, edad y situación socioeconómica.

Se encontraron seis retos principales: estructurar los equipos y seleccionar los sitios; preparar un protocolo único; obtener las aprobaciones éticas; terminar simultáneamente el trabajo sobre el terreno; velar por la calidad de los datos y extraer datos y mantener la uniformidad en todas las bases de datos. Las enseñanzas extraídas muestran que la armonización, los estudios piloto, la uniformidad de los procedimientos, el riguroso control de la calidad de los datos y la comunicación y colaboración entre todos los sitios son imperativos. Los obstáculos incluyeron la complejidad de la organización, el reclutamiento de colaboradores y personal de investigación, la cooperación institucional, el desarrollo de infraestructura y la definición de los recursos. Antes de comenzar el trabajo sobre el terreno, se debe llegar a un consenso acerca de mediciones y resultados uniformes y la metodología de recopilación de datos, así como un plan para la gestión y el análisis de los datos y la comunicación, publicación y difusión de los resultados del estudio. A pesar de que estos estudios constituyen un desafío, hacen posible establecer una base científica para los estudios sobre la nutrición, la actividad física y otros temas de salud, al facilitar las comparaciones entre los países de América Latina.

## RESUMO

Neste relato se examinam os desafios de realizar um estudo transversal multicêntrico em países com culturas diversas e os ensinamentos tirados. O Estudo Latino-americano de Nutrição e Saúde (ELANS) serviu de estudo de viabilidade, englobando as cidades mais populosas de oito países da América Latina (Argentina, Brasil, Chile, Colômbia, Costa Rica, Equador, Peru e Venezuela) em 2014–2015, representando cerca de 40% da população das Américas. A amostra pretendida compreendeu 9.000 indivíduos com 15 a 65 anos de idade e foi estratificada por localização geográfica (exclusivamente áreas urbanas), sexo, idade e nível socioeconômico.

Os seis principais desafios identificados foram: estruturar as equipes e selecionar as sedes, elaborar um único protocolo, obter as aprovações dos comitês de ética, realizar trabalho de campo simultâneo, assegurar a qualidade dos dados e extrair os dados e manter a consistência em todas as bases de dados. Os ensinamentos tirados demonstram serem imprescindíveis harmonização, estudo-piloto, uniformidade dos procedimentos, ótimo controle da qualidade dos dados e comunicação e colaboração entre as sedes. Entre as barreiras estão a complexidade organizacional, recrutamento de colaboradores e pessoal de pesquisa, cooperação institucional, desenvolvimento de infraestrutura e identificação dos recursos. Antes do início do trabalho de campo, deve-se chegar a um consenso sobre medidas e resultados uniformes e metodologia de coleta de dados assim como um plano para o gerenciamento e análise dos dados, comunicação, publicação e disseminação dos resultados dos estudos. Apesar da sua complexidade, tais estudos têm um grande potencial de fundar uma base científica para estudos de nutrição, atividade física e outros tópicos relacionados à saúde, ao mesmo tempo que facilitam comparações entre os países da América Latina.

Growing interest in community-based public health and policy interventions that reduce obesity and improve nutrition has prompted multicenter studies to understand population-based behaviors and outcomes. In contrast to multi-site studies whose main purpose is to obtain a larger sample, multicenter studies are designed jointly by Principal Investigators (PIs) at all sites. This means that all are involved in planning the study protocol and procedures, are scientifically responsible for the study results, and participate actively in manuscripts and other dissemination activities. These studies have been increasingly valued due to large sample sizes, ability to explore differences across sites, and the increased generalization of results (1). Multicenter collaborations also allow recruitment of more diverse populations, within a much shorter time frame. While challenging, such studies offer great potential for building a scientific base for the study of obesity and for planning health policies and intervention programs. However, such studies require heightened attention to detail, simultaneity, comprehensive planning, and collaboration with colleagues (2).

Several large, multicenter, observational studies have been conducted to investigate the nutritional and physical activity status of various populations (3 – 6). The majority of these studies have been performed separately by each country and unified later. In most cases, differing methodologies were used to assess food consumption and physical activity, or samples of each country were not representative of specific populations. In Latin America, few studies have been conducted that represent the reality of each country and region.

The Latin American Study of Nutrition and Health (ELANS), however, is a cross-sectional, multicenter study, that was conducted simultaneously in the urban populations of the most populous cities of eight countries in Latin America. This paper highlights the principle lessons learned from the ELANS, including the key decisions, challenges and barriers, and logistical strengths and limitations encountered during the design process, data collection, and data entry.

## SURVEY METHODOLOGY

ELANS is a household-based, multinational, cross-sectional survey that aims to describe the nutritional status of people in Latin America and to investigate food and nutrient intake, as well as physical activity levels among representative samples of urban populations. The total population of the eight study countries—Argentina, Brazil, Chile, Colombia, Costa Rica, Ecuador, Peru, and Venezuela—represents about 40% of the population of the Americas. The target sample included 9 000 individuals, 15 – 65 years of age, and was stratified by geographic location (only urban areas), gender, age, and socioeconomic status. The rationale and design of the study are reported in more detail elsewhere (7). In brief, the ELANS protocol was designed to collect data at the individual level using questionnaires (sociodemographic, dietary intake, and physical activity) and objective measurements (accelerometry and anthropometry).

## CHALLENGE 1: STRUCTURING A TEAM AND SELECTING SITES

Selected ELANS countries differed in demographic and some socioeconomic indicators, reflecting the differences present in Latin America (Table 1).

**TABLE 1.** Site characteristics of the eight countries participating in the Latin American Study of Nutrition and Health, 2013 - 2014

	Argentina	Brazil	Chile	Colombia	Costa Rica	Ecuador	Peru	Venezuela
Population size^a^	43 132 000	205 620 000	18 006 000	48 584 000	4 832 000	16 205 000	30 814 175	30 620 000
Urban population^b^	92%	85%	89%	76%	76%	64%	76%	89%
GDP^c^	12 509	11 384	14 528	7 903	10 415	6 345	6 541	12 771^f^
GINI index^d^ (22)	42,3	52,9	50,5	53,5	49,2	47,3	44,7	46,9^g^
Obesity in adults^e^	26,3%	20%	27,8%	21%	24.3%	18,7%	21,1%	24,8%

***Source:*** Prepared by the authors based on the study literature.

a Census reports from the country’s national statistical offices.

b Percent of the country’s total population (22).

c Gross Domestic Product per capita in current US$ (23).

d Measure of statistical dispersion, intended to measure the distribution of wealth in a country (21).

e Body Mass Index ≥ 30 (24).

f World Bank 2012; all others, 2014.

g 2006; all others, 2013.

Key factors in the ELANS project implementation were the selection of team members and the inclusion of multidisciplinary professional, technical, and scientific advisors with complementary expertise in epidemiology, energy balance, physical activity, and statistics. PIs were selected based on their ability to conduct epidemiological studies (investigator fieldwork experience, research background, and academic support structure for research) and diversity of geographic location in Latin America.

**FIGURE 1.**
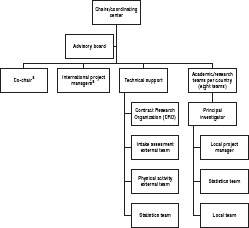
Organizational structure of Latin American Study of Nutrition and Health Project, 2016

^a^International team composed by chairs/coordinating center, co-chair and international project managers.

***Source:*** Prepared by the authors based on the project survey.

All of the academic/research teams were composed of researchers, undergraduates, and postgraduate health science students based in universities or in association with universities, with the exception of one based at a research institute (Table 2). The organizational structure had three branches: academic team, technical support team (physical activity and food intake assessment, anthropometrics), and operational team (Figure 1). The organizational structure of the ELANS was defined during the first design meetings among the researchers, including the scope of the study, the complexity of communications, and what agreements would have to be reached. The study management and quality control strategies are described elsewhere (7).

It is important to highlight that project coordination was centralized in an international team composed of two chairpersons, a co-chair, and two international project managers. This centralized approach ensured that any barriers that arose would be resolved rapidly, that communication among all teams would be effective, and that research uniformity would be maintained. By bringing together expert researchers in the field of physical activity and statistics, the ELANS gained greater detail and depth. Additionally, an external advisory board experienced with similar epidemiological studies provided decision-making guidance required to continue moving the project forward.

**TABLE 2.** Fieldwork characteristics in the Latin American Study of Nutrition and Health, 2014 - 2016

	Argentina	Brazil	Chile	Colombia	Costa Rica	Ecuador	Peru	Venezuela
Study name	Estudio Argentino de Nutrición y Salud	Estudo Brasileiro de Nutriçao e Saude	Estudio Latino Americano de Nutrición y Salud-Chile	Estudio Latino Americano de Nutrición y Salud-Colombia	Análisis del balance energetico y factores de riesgo de obesidad en la población costarricense	El estudio del balance enérgico de una muestra poblacional, Ecuador	Estudio Latino Americano de Nutrición y Salud-Perú	Estudio Latino Americano de Nutrición y Salud-Venezuela
Type of institution	Research institution and university	Private children’s hospital and federal university	University	University	University	University	Research institution	Research institution and university
Number of researchers on team	7	7	10	5	3	3	12	12
Number of cities evaluated	12	23	9	11	7	9	10	13
*n* participants (total; accelerometer)	1 200; 319	2 000; 569	870; 308	1 230; 405	790; 282	800; 268	1 100; 360	1 134; 368
Administration mode	Tablet	Tablet	Tablet	Tablet	Tablet	Paper	Tablet	Paper
Fieldwork period	October 2014 – July 2015	October 2014 – July 2015	September 2014 – July 2015	October 2014 – July 2015	November 2014 – July 2015	September 2014 – July 2015	November 2014 – May 2015	March 2015 – February 2016
Number of fieldwork interviewers	45	40	25	42	13	22	36	36

***Source:*** Prepared by the authors based on the project survey.

### Lessons learned:

- A collaborative, multidisciplinary team of experts with extensive scientific experience and effective communication tools is essential.
- Supervision by an international team is crucial. The appointment of an experienced and proactive coordinating investigator to mentor and counsel other sites ensures consistency and support where it is needed.
- Designating/assigning an international project manager who provides overarching communication and can ensure issues are quickly detected and measures are continuously improved.

## CHALLENGE 2. DEVELOPING AND IMPLEMENTING A SINGLE STANDARDIZED PROTOCOL

At the start, when the decision to collect common data across the sites was made, it became apparent that a single protocol and a uniform set of data collection tools would be required. The process of creating a single protocol involved 15 months of collaborative work with in-person meetings and conference calls with the PIs. All decisions were guided by scientific evidence and fieldwork logistics. The advice from experienced external advisors with differing expertise was important.

Considering the large number of sites, eight in this case, a tools standardization process was developed to ensure equal standards for data collection (8) at every site. The choice of dietary method used in the ELANS was based on existing survey studies, such as NHANES (9) and HELENA (3), as well as the experience of some of the PIs. Repeated 24-hour recalls (24hr) are considered useful tools in providing national-and group-level estimates of usual dietary intakes of individuals, as well as in describing usual intake distributions of populations, using appropriate statistical approaches and controlling for intra-individual variability.

The standardization of procedures for 24hr administration and analysis was necessary to ensure equivalence of the dietary outcomes across participating centers. The Multiple Pass Method (10) was unanimously chosen. All PIs agreed to use the Nutrition Data System for Research software (Minnesota University, Minneapolis, Minnesota, United States; NDSR) to allow ELANS inter-country comparisons. An extensive process of harmonization was initiated, as described elsewhere (11).

The International Physical Activity Questionnaire has been validated in countries of Latin America; however, the Mexican (Spanish) version, (12) adapted by the International Study of Physical Activity and Built Environments was selected for use after cultural adaptations for wording and examples (13, 14).

The objective measure of physical activity was a crucial aspect of the ELANS that provided accurate estimates of physical activity and energy expenditure in this Latin American population. Due to logistic and financial matters, efforts were made to ensure that a range of 25% – 40% of each sample would wear the accelerometer for 7 days.

A major concern was that all sites should use the same or equally-reliable equipment validated by previous studies. It is noteworthy that the PIs faced many problems with the purchase of imported equipment (e.g., accelerometers, scales, stadiometers). Due to customs and mail delivery problems, all sites had to delay the start of the fieldwork.

The socioeconomic questions used in ELANS were designed based on questionnaires used by each national statistics office, or otherwise used most frequently by each country (15 – 20). Due to differing variables used across countries to determine Socioeconomic Levels (SEL), a rule was developed to equate the different classification systems of the eight countries (Table 3). Based on this, three levels of classification were established for SEL and included equivalent characteristics for all countries: high, medium, and low. The same procedure was performed to establish equalization across levels of educational in the eight countries, and also resulted in three classifications: primary to incomplete secondary schooling; complete secondary to incomplete higher/tertiary education (technical/university); and complete higher education (technical/university).

**TABLE 3.** Socioeconomic and educational level equalization between eight countries participating in the Latin American Study of Nutrition and Health, 2016

	Argentina	Brazil	Chile	Colombia	Costa Rica	Ecuador	Peru	Venezuela
**Socioeconomic level**^**a**^								
High	A, B, C1	A1, A2, B1	A, B, C1	5/6	A, B, C1	A, B	A	A, B, C+
Medium	C2, C3	B2, C1	C2, C3	3/4	C2, C3	C+	B, C	C
Low	D1, D2	C2, D, E	D, E	1/2	D1, D2	C-, D	D, E	D, E
**Educational level**								
Primary – incomplete secondary schooling	– Illiterate – Complete or incomplete primary – Incomplete secondary	– Illiterate – Complete or incomplete fundamental – Incomplete secondary	– Illiterate – Complete or incomplete basic – Incomplete humanistic – Incomplete middle	– Illiterate – Incomplete primary or baccalaureate – Incomplete secondary	– Illiterate – Complete or incomplete primary – Incomplete secondary	– Illiterate – Complete or incomplete primary – Incomplete secondary – Incomplete baccalaureate	– Illiterate – Complete or incomplete primary – Incomplete secondary	– Illiterate – Complete or incomplete basic
Complete secondary – incomplete higher/tertiary education	– Complete secondary – Incomplete tertiary – Incomplete university	– Complete secondary – Incomplete higher education	– Complete humanistic – Complete middle – Incomplete professional-technical – Incomplete bachelor	– Complete baccalaureate – Incomplete technical – Incomplete university	– Complete secondary or vocational – Incomplete university	– Complete secondary or baccalaureate – Incomplete university	– Complete secondary – Incomplete university	– Complete middle and diversified
Complete higher education	– Complete tertiary – Complete university – Post-graduate	– Complete higher education	– Complete professionaltechnical – Complete bachelor – Complete school of non-commissioned officers – Post-graduate	– Complete technical or technological – Complete university – Specialization, masters or doctorate	– Complete university – Masters or doctorate	– More years of superior education (without post-graduate) – Complete university – Post-graduate	– Complete technical – Complete university – Post-graduate	– Complete technical – Complete university – Doctorate

***Source:*** Prepared by the authors based on the project survey.

a Classifications according to those provided by National Institutes of Statistics of each country.

### Lessons learned:

- Input from local teams is essential during protocol development and planning of field work to ensure achievable goals.
- Developing a feasible and realistic protocol may be more difficult than first expected. Standardization and harmonization of foods in software require extensive and innovative work. The use of accelerometers requires caution, as compliance is difficult to achieve. Measurement and definitions of socioeconomic status and educational level across countries require close attention to ensure comparability.
- Equipment availability and procurement in different countries requires prior planning and ample time. Possible delivery delays and customs tariffs should be included in the schedule and budget.

## CHALLENGE 3. OBTAINING ETHICS APPROVALS

The process for ethical reviews and timeframes for obtaining approvals vary greatly among countries. To address this challenge and to reduce the burden of conducting multiple local ethics reviews, an external institutional review board (IRB) was selected to perform a centralized review of the protocol, the informed consent template, and other study documents. In this case, Western IRB (WIRB, Puyallup, Washington, United States) was chosen as an independent international organization. However, to obtain WIRB approval, each site also had to submit the protocol and informed consent through a local IRB. After approval of local IRBs, ELANS received WIRB approval.

### Lesson learned:

- Obtaining ethics approvals for a multicenter study must be carefully planned and, preferably, centralized, with extra time allotted for reviews at multiple levels.

## CHALLENGE 4. ACCOMPLISHING SIMULTAENOUS FIELDWORK

During the common protocol design, the complexity of the logistics—a large number of interviewers performing field data collection simultaneously at differing sites—it became apparent that a standardized and uniform application of questionnaires and measurements was needed. These led to the decision to use a Contract Research Organization (CRO) with offices in all eight countries involved in ELANS. This CRO was responsible for all participant sampling and recruitment and data collection and entry, except for dietary recalls, which were managed by each local institution. All procedures were continuously monitored by the international team, and a weekly meeting of PIs was held throughout the fieldwork.

Frequent contact with field supervisors was necessary to ensure quality data collection. At the start, there were many mistakes due to the interviewers’ lack of experience; later, there seemed to be less attention to detail. To accomplish the data collection on time, local interviewers were hired and trained to work in different parts of each country simultaneously. Interviewers with previous health research experience produced better quality interviews. Faster fieldwork was experienced in some countries, such as Chile, where interviewers were health science students.

Standardized training and operating manuals were designed for use in all countries; however, revisions by the international team were made on an as-needed basis to ensure equivalent measures, described elsewhere (7). Three operating manuals provided well-structured guidance for interviewers on anthropometric measurements, dietary intake recalls, and accelerometer use and preparation. A fourth operating manual was designed to ensure proper use of the NDSR software by researchers working with the dietary data. The interviewers’ work was continuously supervised during the data collection period.

To ensure functionality of data collection procedures, a pilot study was performed before fieldwork began. It tested the tools and accompanying procedures at all the sites and identified performance differences across them (8). One of the main difficulties during the pilot study was a refusal to participate, due either to time constraints, lack of interest, or fear of strangers. To overcome this, several strategies were used: leaflets on the ELANS project were distributed in the neighborhoods prior to selection of households; interviewers showed an official identification badge to prospective participants and wore an identifying apron; and during the first visit, an informative letter, including the researchers’ contact information, was provided to each participant. Other issues identified during the pilot study were discussed and corrected, as needed.

### Lessons learned:

- A pilot study is important to ensuring feasibility, efficiency, and adherence to protocols and procedures. Although it delayed the study start, the pilot proved to be important in preventing measurement errors and improving participant compliance.
- Thorough training is fundamental for interviewers and ample time should be allotted for this purpose. Interviewers with experience or training in health sciences are preferable.
- Continuous supervision of interviewers’ tasks is critical for maintaining the data quality of the fieldwork.
- A good relationship and open communication between the academic/research team and the CRO are key to conducting efficient supervision and monitoring procedures.

## CHALLENGE 5. ENSURING DATA QUALITY

Data reliability and credibility are essential to the success of ELANS. Threats to data quality were identified during the protocol design process, the pilot study, and the first weeks of the fieldwork. Thus, the coordinating investigators/chairs proposed adopting procedures in all sites to ensure high-quality data and reliable information. These included preparatory meetings; detailed operating manuals; site visits; technical visits to participating centers; interviewer training; close monitoring of data collection and data entry; retraining of interviewers when needed; concurrent query management and fieldwork supervision; partial database generation; inconsistency checks of anthropometric, physical activity, and food intake data; and when possible, a return visit to the participant’s household to correct unclear, incomplete, or questionable responses.

Monthly, each center sent information on the status of interviews, entry of 24hr food records in NDSR, and the accelerometer files, plus reported any difficulties or impediments to the coordinating center. It is worth noting that considerable effort went into food-matching between local foods/recipes and the foods available in the NDSR database, as described by Kovalskys and colleagues (11).

### Lessons learned:

- Maintaining regular, study-wide communication among PIs, the CRO, and the accelerometer, nutritional, and statistical centers is critical to data quality.

## CHALLENGE 6. EXTRACTING DATA AND DATABASE CONSISTENCY

In all countries, except Ecuador and Venezuela, the CRO had computer tablets available for data collection. When the electronic form was created, internal consistency checks were already programmed into the software to alert the interviewers to outliers and other issues during real-time data entry.

**FIGURE 2.**
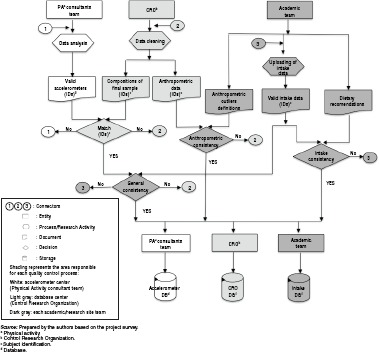
Flowchart for fieldwork closure in the Latin American Study of Nutrition and Health, 2016

***Source:*** Prepared by the authors based on the project survey.

^a^ Physical activity.

^b^ Control Research Organization.

^c^ Subject identification.

^d^ Database.

Throughout the fieldwork, data quality-control was carried out periodically. The types of inconsistencies that were found led the team to develop a second phase of consistency-checking post data collection. Each academic/research site team was responsible for this second phase. A specified procedure was used to detect possible errors of data consistency before the generation of each final database (Figure 2). All data collected were simultaneously reviewed by the CRO, the accelerometer center and its Physical Activity consultant team (PA), and each academic/research site team. The PA consultants and each academic/research team performed participant identification (ID)-matching between their databases (accelerometer database and food intake database, respectively) and the CRO database. Additionally, each academic/research team conducted anthropometric and dietary intake consistency checks. Upon successful completion, a general consistency check was then performed by each academic/research team. Thereafter, final versions of the datasets were generated by the PA consultants, CRO, and academic/research team. If any step was not successfully concluded during this process, it was restarted, as shown in Figure 2 by connectors 1, 2, and 3. If any participant needed to be excluded from the sample, the academic team informed the CRO, and the case was replaced with a matched case—same gender, age group, SEL, and geographic area—from the oversample.

Common sources of errors included simple typographical errors; consistency errors, such as birth date and age did not concur; differing participant ID and/or demographic information between first and second visit; insufficient accelerometer wear time (hours/day and/or number of days); programming or accelerometer malfunctions; or absence of PA logs. After identifying the source of each error, most could be resolved by communication between the centers. If the entry was unclear, missing, or otherwise suspicious, field staff were contacted for correction or verification at participating households. Accelerometer data errors were corrected in the office when possible; when not, the individuals were moved to the group without accelerometer use (60% of the sample). When this group was full, the rest were assigned to the oversample group. As a considerable number of cases were found to be invalid due to insufficient wear time, programming error, or device malfunction, the final percentage was reduced to 25% of the sample.

Each site was responsible for verifying the quality of the data registered in its 24hr food records, and as needed, field staff were contacted for correction or verification at participants’ households. Several steps were developed for analysis of dietary intake data consistent with NDSR. This was a difficult phase that required validation from several researchers. Technical support and guidance were required by all the research teams. Special consideration was given to the variations in fortified local foods and processed food products, and to correcting micronutrient contents of foods according to local food composition tables. All of the researchers provided input for this process and approved the final version of the resulting document.

### Lessons learned:

- Extensive expertise in dietary assessment among the researchers is important because this sensitive part of the data collection required the greatest number of corrections to inconsistencies.
- Data cleaning demands close attention to detail and collaboration of multiple stakeholders.
- Correct use of accelerometers relies on thorough interviewer training so that they can, in turn, effectively instruct participants. Timely delivery of data to the PA team by the CRO is needed for corrections to be made. A 70% accelerometer compliance rate should be taken into account, with fewer valid versus measured cases in the final sample.
- A data analysis and publication plan that ensures appropriate data management and dissemination must be developed with input from all team members and be followed meticulously through the end of the study.

## FINAL CONSIDERATIONS

The development of a multicenter household cross-sectional survey of the nutritional and physical activity status of adolescents and adults in eight countries of Latin America was an innovative and fundamental step toward better understanding behaviors and their relationship to health in the region. This survey contributed to several lessons learned regarding the organization of a multicenter study. The feasibility of performing such a study depends on many considerations, including standardization of data collection, maintenance of high data quality, and collaboration across sites. Among the greatest barriers to conducting studies such as this one are the inherent organizational complexity, recruitment of collaborators and research staff, institutional cooperation, development of infrastructure, and identification of resources. By implementing the various strategies detailed, this project overcame various challenges to ensure the integrity of its cross-site data collection.

The entire research team at each participating center and the coordinating center—the PIs, local investigators, consultants, coordinators, research assistants, data managers, system analysts, statisticians, network managers, and accountants—proved to be essential to study development and performance. Ideas and solutions, as well as planning for analysis and interpretation of results, arose naturally when coming from a team thinking and working together. The institutions involved all benefited from the exchange of knowledge among researchers, the equipment acquisitions, the use of internationally-recognized methodology, and wider study dissemination.

Multicenter cooperative studies while challenging, offer great potential for building a scientific base for studies on nutrition and health. Considering the methods and experience of other multicenter studies while developing the study design of the ELANS was important for surmounting possible obstacles and allowing faster progress. To this end, the lessons learned during ELANS provide new perspectives for better planning of financial, staff, and technological resources for similar future studies.

### Acknowledgements.

The authors would like to thank the following individuals at each of the participating sites who made substantial contributions to the ELANS: Luis Costa, Regina Fisberg, Alejandra Guidi, Mariela Jauregui, Beate Lloyd, Brenda Lynch, and Bruno Zoca de Oliveira.

The following are members of ELANS Study Group:

*Chairpersons:* Mauro Fisberg and Irina Kovalskys.

*Co-chair:* Georgina Gómez Salas.

*Core Group members:* Lilia Yadira Cortés Sanabria, Mauro Fisberg, Georgina Gómez Salas, Marianella Herrera-Cuenca, Irina Kovalskys, Rossina Gabriella Pareja Torres, Attilio Rigotti, and Martha Cecilia Yépez García.

*External advisory board:* Berthold Koletzko, Luis A. Moreno, Michael Pratt, and Katherine L. Tucker.

*Project Managers:* Viviana Guajardo and Ioná Zalcman Zimberg.

*International Life Sciences Institute, Argentina:* María Paz Amigo, Fernando Cardini, Viviana Guajardo, Ximena Janezic, and Irina Kovalskys.

*Instituto Pensi–Hospital Infantil Sabara, Brazil:* Natasha Aparecida Grande de França, Mauro Fisberg, Agatha Nogueira Previdelli, and Ioná Zalcman Zimberg.

*Pontificia Universidad Catolica de Chile:* Óscar Castillo, Guadalupe Echeverría, Leslie Landaeta, and Attilio Rigotti.

*Pontificia Universidad Javeriana, Colombia:* Yuri Milena Castillo, Lilia Yadira Cortés Sanabria, Luisa Fernanda Tobar, and Luz Nayibe Vargas.

*Universidad de Costa Rica, Costa Rica:* Anne Chinnock, Georgina Gómez Salas, and Rafael Monge Rojas.

*Universidad San Francisco de Quito, Ecuador:* Lucia Eguiguren, Mónica Villar Cáceres, and Martha Cecilia Yépez García.

*Instituto de Investigación Nutricional, Peru:* Mellisa Abad, Maria Reyna Liria, Krysty Meza, Rossina Pareja Torres, and Mary Penny.

*Universidad Central de Venezuela:* Pablo Hernández, Marianella Herrera-Cuenca, Maritza Landaeta, Betty Méndez, Guillermo Ramírez, and Maura Vasquez.

*Accelerometry analysis:* Claudia Alberico and Priscila Bezerra Gonçalves.

*Physical activity advisor:* Gerson Luis de Moraes Ferrari.

*Dietary intake advisor:* Ágatha Nogueira Previdelli.

### **Funding**.

The ELANS is supported by a scientific grant from the Coca Cola Company (Atlanta, Georgia, United States) and by grants and/or support from the Instituto Pensi/Hospital Infantil Sabara, International Life Science Institute of Argentina, Universidad de Costa Rica, Pontificia Universidad Católica de Chile, Pontificia Universidad Javeriana, Universidad Central de Venezuela/Fundación Bengoa, Universidad San Francisco de Quito, and Instituto de Investigación Nutricional de Perú. The funders had no role in study design, data collection, analysis, the decision to publish, or the preparation of this manuscript. MF is a member of the Board of Directors of Danone Institute International (Paris, France). KLT received consulting fees from the Coca Cola Company to participate.

### **Disclaimer**.

Authors hold sole responsibility for the views expressed in the manuscript, which may not necessarily reflect the opinion or policy of the *RPSP/PAJPH* and/or PAHO.
